# Clinical features of febrile seizures in children with COVID-19: an observational study from a tertiary care hospital in China

**DOI:** 10.3389/fped.2023.1290806

**Published:** 2023-10-06

**Authors:** Cuiyun Fang, Yuan Zhou, Wei Fan, Chunsheng Zhang, Yi Yang

**Affiliations:** ^1^ Department of Nursing, Liyang People’s Hospital, Liyang, China; ^2^ Department of Pediatrics, Liyang People’s Hospital, Liyang, China

**Keywords:** clinical features, febrile seizures, children, COVID-19, disease burden

## Abstract

**Background:**

Febrile seizures are a common neurologic manifestation in children with coronavirus disease 2019 (COVID-19). Compared to seasonal respiratory viruses, severe acute respiratory syndrome coronavirus 2 (SARS-CoV-2) has a pronounced neurological impact, with the result that febrile seizures with COVID-19 may exhibit unique clinical features.

**Materials and methods:**

We conducted a retrospective study in a tertiary care hospital in China. We collected medical record information on febrile seizures with COVID-19, including demographic characteristics, clinical features, laboratory tests, and disease burden. Subsequently, the data were then analyzed descriptively.

**Results:**

A total of 103 children diagnosed with febrile seizures and positive COVID-19 PCR results were included in our study. Among them, 81 (78.6%) were males and 22 (21.4%) were females. The age of onset of febrile seizures ranged from 14 to 57 months, with a mean age of 34.9 ± 6.24 months. Complex febrile seizures were observed in 34 (33%) cases. Antiseizure medications were administered to 24 (23.3%) patients. Laboratory tests showed a white blood cell count of (27.05 ± 8.20) × 10^3^/µl, a neutrophil count of (20.09 ± 5.66) × 10^3^/µl and a lymphocyte count of (6.44 ± 1.86) × 10^3^/µl. A creatine kinase level was significantly elevated, with a mean value of (412.00 ± 158.96) U/L. The mean length of stay was 4.36 days. Twelve patients (11.7%) required intensive care services, but there were no deaths or patients remaining on antiseizure medications after discharge.

**Conclusion:**

In the post-epidemic era of COVID-19, pediatric clinicians should be aware of the changing clinical features of febrile seizures associated with COVID-19. The average age of onset has increased, with a higher proportion of males. Length of stay and hospitalization costs did not increase significantly. The prognosis remained favorable, although a small number of children required intensive care services during the acute phase.

## 1. Introduction

Febrile seizures (FS) are the most common seizure disorder in children, defined as seizures accompanied by fever (temperature ≥38°C) without central nervous system infection (1). Typically, febrile seizures manifest in children between the ages of 6 months and 5 years. The prevalence of febrile seizures in the United States and Western Europe ranges from 2% to 5%, while in Japan, it is estimated to be between 6% and 9% (2). Febrile seizures have been categorized as simple febrile seizures and complex febrile seizures. Simple febrile seizures are usually considered to have a good prognosis, whereas complex febrile seizures are associated with recurrent febrile seizures, epilepsy, and neurologic deficits (3, 4). There is a genetic predisposition to febrile seizures. The risk of developing a febrile seizure is approximately 20% when a sibling is affected, and this risk increases to 33% when both parents are affected (5). At present, the exact cause of febrile seizures remains unknown, but it is most commonly associated with fever resulting from viral and bacterial infections, as well as fever resulting from various vaccinations (6). Risk factors for febrile seizures include a family history of febrile seizures, prenatal and postnatal complications, the rate of elevated body temperature, microcytic hypochromic anemia, deficiencies in iron and zinc, as well as low serum levels of calcium, sodium, and glucose (5). Viruses commonly associated with febrile seizures included human herpesvirus 6, influenza virus, adenovirus, parainfluenza virus, varicella virus, respiratory syncytial virus, and rotavirus (7).

Coronavirus disease 2019 (COVID-19) is a severe infectious disease caused by the severe acute respiratory syndrome coronavirus 2 (SARS-CoV-2). Currently, as a result of the high levels of population immunity to SARS-CoV-2, countries have implemented relaxed restrictions on COVID-19 (8). While SARS-CoV-2 primarily affects the respiratory system, it frequently involves the nervous system and presents with various manifestations (9). In some cases, especially in the setting of multisystem inflammatory syndrome in children (MIS-C), life-threatening neurologic involvement may occur in children. Febrile seizures, a common neurologic manifestation in children with COVID-19, have significantly increased during the omicron outbreak (10, 11). SARS-CoV-2 has a significant impact on the nervous system. As a result, febrile seizures with COVID-19 may exhibit unique clinical features compared to patients with seasonal viral infections. Furthermore, limited literature has reported on the disease burden of febrile seizures with COVID-19. Therefore, we conducted this retrospective study to characterize the clinical features and disease burden of febrile seizures in patients with COVID-19.

## 2. Materials and methods

### 2.1. Study design

This is a retrospective descriptive study. We comprehensively searched the medical records of all patients diagnosed with febrile seizures who also had positive results of COVID-19 polymerase chain reaction (PCR) tests from January 2020 to June 2023 in Liyang People's Hospital through an electronic case system. Liyang People's Hospital is the only tertiary hospital in the area.

A simple febrile seizure is defined as a primary generalized seizure lasting no more than 15 min and not recurring within 24 h. It occurs during a fever that is not due to an acute neurologic illness. The age of onset is between 6 months and 5 years, and there are no neurologic deficits. Fever may not be detected prior to the seizure, but must be present at least immediately after the acute seizure (12). A complex febrile seizure is defined as focal, of long duration (≥15 min), and/or recurring within 24 h. It is associated with postictal neurologic abnormalities, more commonly postictal paralysis (Todd's palsy), or with prior neurologic deficits. It also includes children with prolonged seizures stopped due to antiseizure medication by the 15th minute (13). Status epilepticus is defined as seizure duration ≥30 min or recurrent seizures, interictal consciousness not recovered in 30 min or more.

The following patients were excluded: previous non-febrile seizures, epilepsy, brain imaging findings that could lead to epilepsy, and incomplete medical records. If a patient was hospitalized multiple times during the study period, medical record information was collected only for the last hospitalization.

Demographic characteristics, including age, gender, history of febrile seizures, and family history of febrile seizures were collected. We also collected clinical features including peak fever (the highest temperature recorded during the course of the disease), duration of fever, interval between fever and seizures, number, duration and type of seizures, and antiseizure medications. Immediately after hospitalization (usually within a few hours), laboratory tests were performed, including white blood cells (WBC), neutrophils (NE), lymphocytes (LY), hemoglobin (HB), platelets (PLT), C-reactive protein (CRP), procalcitonin (PCT), electrolytes, aspartate aminotransferase (AST), alanine aminotransferase (ALT), creatine kinase (CK). Brain tests including electroencephalogram (EEG) and brain computed tomography (CT) results were also collected. In addition, information was collected on the burden of disease, including length of stay, hospitalization costs, and admission to the intensive care unit.

### 2.2. Statistical analysis

Categorical variables were expressed as *n* (%) and were compared using the chi-square test, Fisher's exact test, or continuity correction. Data for continuous variables were expressed as mean ± standard deviation. Normally distributed data were analyzed for significance using the *t*-test, while non-normally distributed data were analyzed using the Wilcoxon rank sum test. All tests were considered statistically significant at two-sided *P* < 0.05. All statistical analyses were performed using SPSS software (version 20.0; SPSS, Chicago, IL, USA).

## 3. Results

### 3.1. General information

During the study, 117 children with febrile seizures and positive COVID-19 PCR results were enrolled. According to our exclusion criteria, 14 patients were excluded, leaving a total of 103 patients in this study. Table 1 summarizes the demographic characteristics of the study population. There were 81 (78.6%) males and 22 (21.4%) females among them. With a male-to-female ratio of 3.68:1, the proportion of males was significantly higher than that of females. The age of onset of febrile seizures ranged from 14 to 57 months, with a mean age of 34.9 ± 6.24 months. There was a history of febrile seizures in 45 (43.7%) patients and a family history of febrile seizures in 6 (5.8%) patients.

**Table 1:** The demographic characteristics of the study patients.

Categories	Value
Sex, male	81 (78.6%)
Age (months)	34.90 ± 6.24
History of FS	45 (43.7%)
Family history of FS	6 (5.8%)

Data are presented as number (%) or mean ± SD values.

SD, standard; FS, febrile seizures.

### 3.2. Clinical features

As for respiratory manifestations, cough was present in 55 (53.4%) cases, nasal congestion in 27 (26.2%), nasal discharge in 22 (21.4%), sore throat in 10 (9.7%), and pneumonia in 2 (1.9%). As shown in Table 2, the average fever peak was 39.4°C, the duration of fever was 2.37 days, and the average time from fever to the onset of seizures was 8.84 h. There were 18 (17.5%) cases of recurrent seizures within 24 h, 6 (5.8%) cases of focal seizures, 14 (13.6%) cases of seizures lasting more than 15 min, and 6(5.8%) cases of status epilepticus. There were 34 (33%) cases of complex febrile seizures. A total of 24 (23.3%) patients received brief antiseizure medications at the time of seizure. Among these, 10 experienced recurrent seizures with short intervals. The initial antiseizure medication administered was chloral hydrate (50 mg/kg, enema), which successfully prevented further recurrence. The remaining 14 patients had seizures lasting more than 15 min, and they were also initially treated with chloral hydrate (50 mg/kg, enema). However, in 6 of these cases, rapid remission did not occur, leading to the administration of a second antiseizure medication. Diazepam (0.3 mg/kg, intravenous) was administered to 5 patients, while phenobarbital (5 mg/kg, intravenous) was given to 1 patient. Following the administration of the second antiseizure medication, all patients experienced resolution of their seizures. Furthermore, we compared the clinical characteristics of male and female children and discovered no significant differences.

**Table 2:** The clinical characteristics of the study patients**.**

Categories	Total	Male	Female	*P*
Fever peak (°C)	39.40 ± 0.53	39.39 ± 0.56	39.38 ± 0.41	0.179
Fever duration (day)	2.37 ± 0.91	2.54 ± 0.84	2.32 ± 1.10	0.066
Interval between fever and seizures (h)	8.84 ± 7.2	8.80 ± 7.08	9.00 ± 8.17	0.512
Recurrent seizures	18 (17.5%)	14 (13.6%)	4 (3.9%)	0.922
Focal seizures	6 (5.8%)	6 (5.8%)	0 (0.0%)	0.188
Seizures lasting ≥15 min	14 (13.6%)	10 (9.7%)	4 (3.9%)	0.479
Status epilepticus	6 (5.8%)	5 (4.9%)	1 (0.9%)	0.773
Complex FS	34 (33%)	26 (25.2%)	8 (7.8%)	0.706
Receiving ASM	24 (23.3%)	18 (17.5%)	6 (5.8%)	0.778

Data are presented as number (%) or mean ± SD values.

SD, standard; FS, Febrile seizure; ASM, antiseizure medications.

### 3.3. Laboratory tests

On laboratory tests, WBC and NE were markedly elevated. The mean value of LY was (6.44 ± 1.86) × 10^3^/µl. PLT, CRP, and PCT were also higher than the upper limit of normal. Also, CK levels were significantly higher. The results of the laboratory tests are shown in Table 3.

**Table 3:** The laboratory tests of the study patients.

Categories	Value
WBC (×10^3^/µl)	27.05 ± 8.20
NE (×10^3^/µl)	20.09 ± 5.66
LY (×10^3^/µl)	6.44 ± 1.86
HB (g/L)	153 ± 125.93
PLT (×10^3^/µl)	779 ± 232.38
CRP (mg/dl)	28.79 ± 5.18
PCT (ng/ml)	6.43 ± 0.59
Na (mmol/L)	142.45 ± 137.42
K (mmol/L)	5.30 ± 4.41
Ca (mmol/L)	2.98 ± 2.46
Cl (mmol/L)	109.97 ± 104.22
CK (U/L)	412.00 ± 158.96
AST (U/L)	81.00 ± 42.20
ALT (U/L)	37.60 ± 19.81

Data are presented as mean ± SD values.

SD, standard; WBC, white blood cells; NE, neutrophil; LY, lymphocyte; HB, hemoglobin; PLT, platelets; CRP, C-reactive protein; PCT, procalcitonin; CK, creatine kinase; ALT, alanine aminotransferase; AST, aspartate aminotransferase.

### 3.4. Brain tests

A total of 85 (82.5%) patients underwent EEG. Among them, 11 (10.7%) had abnormal EEGs showing background slow wave enhancement. A brain CT was performed on 28 (27.2%) and showed no abnormalities. No patient underwent cerebrospinal fluid examination.

### 3.5. Disease burden

As shown in Table 4, the average hospital cost was $258.58 and the average length of stay was 4.36 days. Twelve (11.7%) patients were admitted to the intensive care unit due to unstable vital signs associated with status epilepticus. Once their condition was under control, they were transferred to the general ward. None of the patients died, and none required antiseizure medications after discharge.

**Table 4:** The disease burden of the study patients.

Categories	Value
Hospital costs (dollars)	258.58 ± 72.65
Length of stay (day)	4.36 ± 1.48
Receiving intensive care services	12 (11.7%)

## 4. Discussion

We aimed to comprehensively characterize the clinical features and disease burden of febrile seizures with COVID-19. Our study revealed several important findings. Firstly, we observed that COVID-19 associated febrile seizures had an older age of onset, with a mean age of 34.90 ± 6.24 months. Furthermore, we found a significant difference in gender, with a male-to-female ratio of 3.68: 1. In terms of disease burden, we found that the mean number of days of hospitalization was 4.36 ± 1.48. Additionally, 11.7% of the patients in our study required intensive care services, highlighting the potential severity of febrile seizures with COVID-19. However, it is important to note that no deaths occurred and none of the patients required long-term antiseizure medications after discharge among our study population, indicating a generally positive outcome for these patients.

The neurological effects of COVID-19 have been a source of concern since its pandemic in 2020. According to a recent international multicenter prospective observational study, the most common neurological complications associated with COVID-19 in children was malaise, followed by altered consciousness and myalgia (14). These neurologic complications can occur not only in children with pre-existing underlying conditions but also in previously healthy children (15). While rare, there have been reported cases of neurological complications that can lead to serious or even life-threatening consequences. In a large multicenter study conducted in the United States, 2.5% of children and adolescents hospitalized with acute COVID-19 or MIS-C were reported to present with a range of life-threatening neurological disorders associated with COVID-19, including severe encephalopathy, acute ischemic or hemorrhagic stroke, acute central nervous system infection/acute disseminated encephalomyelitis, acute fulminant cerebral edema, and Guillain Barré syndrome (16). Seizures are also frequently seen in children with COVID-19 and are mostly accompanied by fever. A study involving a large cohort of hospitalized children with COVID-19 reported a prevalence of febrile seizures of 3.9% (10).

In our study, the minimum age of children with febrile seizures with COVID-19 was 14 months and the maximum age was 57 months, with a mean age of 34.9 ± 6.24 months. The mean age of onset of febrile seizures with COVID-19 was atypical, which is in line with the findings of previous studies (17, 18). This is significantly higher than the previously reported mean age of febrile seizures due to other seasonal viral infections, which is 18–20 months (19). Our study also found a significant gender difference in febrile seizures with COVID-19, with a male-to-female ratio of 3.68:1. Gender differences in febrile seizures have been widely reported, but COVID-19 may amplify this difference. Even though males and females are at equal risk for COVID-19 infection, males tend to be associated with more severe complications and outcomes (20–22). Male predominance has been observed in various other complications associated with COVID-19 such as multi-system inflammatory syndrome, myocarditis, and acute respiratory distress syndrome in children (23, 24). Differences in genetic susceptibility, hormonal effects, and immune system response may account for the male bias in COVID-19. Testosterone is hypothesized to have a role in the complicated system that prevents anti-inflammatory cells from participating in the Th1 immune response. Furthermore, estrogen suppresses cell-mediated immune responses by inhibiting proinflammatory *T* cells. In addition, variations in the innate immune systems represented by monocytes and macrophages are proposed as sources of sexual discrepancy in the adaptive immune system (25, 26).

In terms of clinical features, 33% of the patients had complex febrile convulsions, which is higher than previously reported (19). This may be related to the unique neurologic effects of COVID-19. The underlying mechanism by which COVID-19 causes seizures may be related to direct viral damage to nerve cells and inflammation. The neurotoxic and neuroinvasive nature of COVID-19 causes neuronal infection via angiotensin-converting enzyme 2 in cerebral vascular endothelial cells, leading to a cascade of inflammation (27). Inflammation is mediated by cytokines such as Interleukin (IL)-1β, IL-6, Tumor Necrosis Factor-α, and IL-17. These cytokines activate glial cells, leading to a hyperinflammatory response, which increases nervous system excitability and makes children more susceptible to febrile seizures (28). Some seizures that are classified as “febrile seizures” may not actually be febrile seizures, as the virus may have entered the central nervous system. This could partially explain the differing characteristics of these seizures compared to the typical febrile seizures observed thus far. Future studies will be needed to determine if epileptic seizures during a SARS-CoV-2 infection involve the central nervous system, or if they are truly febrile seizures. Children are more prone to seizures than adults, which may be related to a highly excitable nervous system. In addition, due to age-related changes in the blood-brain barrier and neurovascular unit, children and adults respond differently to direct nerve injury by COVID-19 (16).

In laboratory tests, the present study also found a significant increase of CK levels in febrile seizures with COVID-19. Increased CK levels are frequently seen in patients with COVID-19. It has been reported to be associated with the severity of the disease and is a predictor of severe prognosis (29, 30). Increased CK levels have also been reported to be associated with elevated levels of inflammatory factors in patients with COVID-19 (31). The observation that higher biomarkers of inflammation are associated with higher CK values also supports an immune-mediated mechanism for SARS-CoV-2 infection. However, it is unclear whether elevated CK is a virally induced inflammatory response or direct muscle toxicity. Our study found increased WBC and NE in complete blood counts of febrile seizures with COVID-19. Mean WBC in patients with mild COVID-19 have been reported to be in the normal range, with decreased LY and monocyte counts, but WBC and NE can be significantly higher in patients with severe COVID-19, a finding consistent with our study (32). However, the data from the studies we included were only the patients' blood results at the time of admission (usually a short time after the seizure) and lacked the results of ambulatory monitoring. It is obvious that in COVID-19-positive patients, the changes in leukocyte morphology vary in different disease stages, so our findings have limited reference value. COVID-19 did not significantly increase the length of stay in the hospital compared to febrile seizures caused by other viruses (33). Although a small percentage required intensive care services, the overall prognosis was favorable, with no deaths or long-term antiseizure medication requirements. Febrile seizures are typically self-limiting, and the majority of cases do not result in complications. However, they can be highly distressing for families. Pediatricians should prioritize communication with parents of children experiencing febrile seizures and provide guidance on important information, such as managing a new episode (34). This approach can help alleviate caregivers' concerns and prevent the development of inappropriate anxious behaviors associated with “febrile phobia”.

There are also some limitations to this study. First, in our study, only COVID-19 viruses were detected, and it was not possible to determine whether there was a mixture of other viruses, which might have affected our results. In addition, since China's anti-epidemic policy was fully liberalized at a later stage (i.e., during the Omicron period), caution should be exercised in generalizing our findings to all SARS-CoV-2 variants. Finally, our study was a retrospective observational study with an included population of hospitalized patients, which may be subject to recall bias and selection bias. The effect of COVID-19 on febrile seizures in children requires future multicenter studies with large samples.

## 5. Conclusion

In conclusion, our study provides valuable insights into the clinical features and disease burden of febrile seizures associated with COVID-19. The findings suggest an older age of onset, a higher proportion of males, and relatively short hospital stays for these patients. Although a small percentage required intensive care services, the overall prognosis was favorable, with no deaths or long-term antiseizure medication requirements. These findings highlight the importance of understanding and managing febrile seizures in the context of COVID-19, particularly in pediatric populations. Further research is needed to elucidate the underlying mechanisms and risk factors associated with febrile seizures in COVID-19.

## Data Availability

The original contributions presented in the study are included in the article/Supplementary Material, further inquiries can be directed to the corresponding author.
